# Prepandemic Risk Factors of COVID‐19‐Related Concerns in Adolescents During the COVID‐19 Pandemic

**DOI:** 10.1111/jora.12651

**Published:** 2021-08-26

**Authors:** Amanda W. G. van Loon, Hanneke E. Creemers, Simone Vogelaar, Anne C. Miers, Nadira Saab, P. Michiel Westenberg, Jessica J. Asscher

**Affiliations:** ^1^ Utrecht University; ^2^ University of Amsterdam; ^3^ Leiden University

**Keywords:** adolescence, COVID‐19, stress, mental health, longitudinal

## Abstract

To identify adolescents who may be at risk for adverse outcomes, we examined the extent of COVID‐19‐related concerns reported by adolescents and investigated which prepandemic risk and protective factors predicted these concerns during the COVID‐19 pandemic. Dutch adolescents (*N* = 188; *M*_age_ = 13.49, *SD* = .81) were assessed before the pandemic and at eight and ten months into the pandemic. Results demonstrated that adolescents’ most frequently reported COVID‐19‐related concerns were about social activities and getting delayed in school. Adolescents that have specific vulnerabilities before the pandemic (i.e., higher stress, maladaptive coping, or internalizing problems) experience more concerns during the pandemic, stressing the importance of guiding and supporting these adolescents in order to prevent adverse developmental outcomes.

## Introduction

Society at large is heavily affected by the coronavirus disease 2019 (COVID‐19) crisis and the accompanying national government measures to prevent the spread of the virus. However, a hitherto somewhat understudied group in pandemic research is adolescents (Francisco et al., 2020), a group that may be particularly affected by the drastic changes in daily lives since the outbreak of COVID‐19. Adolescence is a period of social development, in which the social environment is increasingly important, especially interactions with peers (Blakemore & Mills, 2014). During this developmental period, adolescents seek independence from their parents and the interaction with and influence of peers increases significantly (de Goede, Branje, de Delsing, & Meeus, 2009). Yet, with social distancing being enforced by the government, the opportunities and possibilities for (social) experiences outside the family home are reduced. In the Netherlands for instance, as of March 15, 2020, public spaces (e.g., schools, restaurants, cinemas, and sport clubs) were closed and public gatherings were prohibited in order to minimize social interactions. As schools were closed and people were strongly advised to work from home, adolescents and their families (e.g., parents and siblings) were forced to spend most of their time at home, together. Moreover, instead of going to school, adolescents had to switch to online education. Although from June 2, 2020, schools gradually reopened (with, in secondary schools, a combination of physical and online education) and other measures were partially lifted (e.g., restaurants and cinemas were allowed to open, as well as sport clubs, yet with a maximized number of visitors to ensure social distancing), other measures were tightened or put into effect (e.g., limiting group sizes and wearing face masks). Moreover, as of December 15, 2020, a hard lockdown was in effect, with public spaces closed, as well as all non‐essential shops. Furthermore, schools were closed again, and students had to switch back to online education (i.e., schools were closed until the end of February 2021 and only partly reopened from March 2021).

The restrictions on adolescents’ daily life and physical peer interactions in particular, the changing demands with regard to school, and the uncertainty about the pandemic and restrictions can cause concern in adolescents. Previous research conducted at the beginning of the COVID‐19 pandemic (i.e., April‐June 2020; in Canada, Australia, and Spain) demonstrated that adolescents between 13 and 18 years old were very concerned about the COVID‐19 pandemic, specifically about school and not being able to see their friends, and to a lesser degree about a loved one becoming ill, finances, and becoming ill themselves (Ellis, Dumas, & Forbes, 2020; Magson et al., 2020; Muñoz‐Fernández & Rodríguez‐Meirinhos, 2021). Girls reported higher COVID‐19 stress levels than boys (Ellis et al., 2020; Muñoz‐Fernández & Rodríguez‐Meirinhos, 2021). Other research indicated that German adolescents aged 11–17 years felt burdened and reported that homeschooling and learning were more difficult at the beginning of the COVID‐19 pandemic (Ravens‐Sieberer et al., 2021). Yet, while some adolescents might experience the COVID‐19 pandemic and accompanied restrictions as very stressful and worrisome, others might adapt quickly to the situation and try to make the most of it (Brailovskaia & Margraf, 2020; Dvorsky, Breaux, & Becker, 2020). How adolescents experience and deal with the COVID‐19 pandemic might have consequences for future challenges, such as school or mental health problems (Wade, Prime, & Browne, 2020). It is, therefore, important to acquire knowledge on prepandemic factors that are associated with higher levels of COVID‐19‐related concerns, to identify adolescents who might need help.

Both negative (i.e., risk) and positive (i.e., protective) prepandemic factors may predict high levels of COVID‐19‐related concerns in adolescents. Since worrying is assumed to play a key role in the development of stress‐related mental health problems (Anniko, Boersma, & Tillfors, 2019), adolescents with elevated symptoms of stress, anxiety, or depression might be especially prone to COVID‐19‐related concerns, as these adolescents tend to worry about situations of which the outcome is uncertain, unpredictable, and potentially negative. A prospective link of prepandemic stress with COVID‐19‐related burden has been identified in adults (Brailovskaia & Margraf, 2020). Furthermore, different coping skills can have negative or positive influences on COVID‐19‐related concerns. Coping behaviors are defined as responses to the demands and emotions caused by stressful events (Lazarus & Folkman, 1984). Research during the first COVID‐19 lockdown demonstrated that negative coping strategies were associated with increases in perceived stress in young Dutch adolescents aged 10 to 13 years (Achterberg, Dobbelaar, Boer, & Crone, 2021). Hence, adolescents that use more maladaptive coping, such as rumination and catastrophizing (e.g., negative ways to cope with stress), might report higher COVID‐19‐related concerns, while adolescents that use more adaptive coping, such as acceptance and positive refocusing (e.g., positive ways to cope with stress), might report lower COVID‐19‐related concerns. Positive well‐being, associated with resilience and adaptive coping (Sagone & Caroli, 2014; Verzeletti, Zammuner, Galli, & Agnoli, 2016), might contribute to fewer concerns about the COVID‐19 pandemic. Indeed, a recent study in adults demonstrated that prepandemic positive mental health was associated with lower levels of COVID‐19‐related burden (Brailovskaia & Margraf, 2020). Finally, social support may prevent escalation of COVID‐19‐related concerns, as research in adolescents showed that social support promoted resilience and well‐being (Liu, Jiang, Li, & Yang, 2021), and adolescents that experienced high levels of social connection reported less distress during the COVID‐19 pandemic (Magson et al., 2020).

Most previous studies about the COVID‐19 pandemic are based on cross‐sectional study designs, lacking information about psychological functioning before the pandemic (Singh et al., 2020; Wade et al., 2020). Moreover, there is limited longitudinal knowledge about the effects of the pandemic in youth (Wade et al., 2020), as its impact may vary over time. To advance previous research, the present study investigated COVID‐19‐related concerns in adolescents at two stages during the pandemic (i.e., at approximately eight and ten months into the pandemic). The first aim was to describe the level of COVID‐19‐related concerns in adolescents during the COVID‐19 pandemic, and to explore gender, age (i.e., school year), or education level differences in these concerns. Moreover, we examined whether COVID‐19‐related concerns changed during the course of the pandemic. The second aim was to investigate which individual prepandemic factors were predictive of COVID‐19‐related concerns during the COVID‐19 pandemic, and to examine predictor differences in type of concerns. It was expected that perceived stress, internalizing problems (i.e., anxiety and depressive symptoms), and maladaptive coping would predict higher levels of COVID‐19‐related concerns, while adaptive coping, well‐being, and social support would predict lower levels of COVID‐19‐related concerns during the COVID‐19‐pandemic. By first assessing the types and levels of concerns among adolescents, and to subsequently predict these by prepandemic factors, we sought to gain insight in this largely unexplored field. It is important to gain longitudinal knowledge about prepandemic factors that affect the experienced level of COVID‐19‐related concerns, as this may help to identify adolescents who might be at risk for later adverse outcomes, such as school or mental health problems (Wade et al., 2020). This may guide professionals (e.g., mental health professionals and school personnel) working with adolescents to provide them with appropriate support.

## Methods

### Study design, participants, and procedure

The data were collected within the context of an ongoing evaluation study conducted in secondary schools in the Netherlands. As the COVID‐19 outbreak occurred during the course of this study, it provided an ideal opportunity to collect data about COVID‐19 related concerns in adolescents. This evaluation study investigated the effectiveness of school‐based skills‐training programs, in which secondary school students enrolled by self‐selection (van Loon et al., 2019). The current sample is a subsample, consisting of all adolescents that were included in the effectiveness study in February 2020. Due to the outbreak of COVID‐19 and the consequent school closing, the school‐based skills‐training programs for which they registered were ended after some initial sessions.

**Figure 1:**
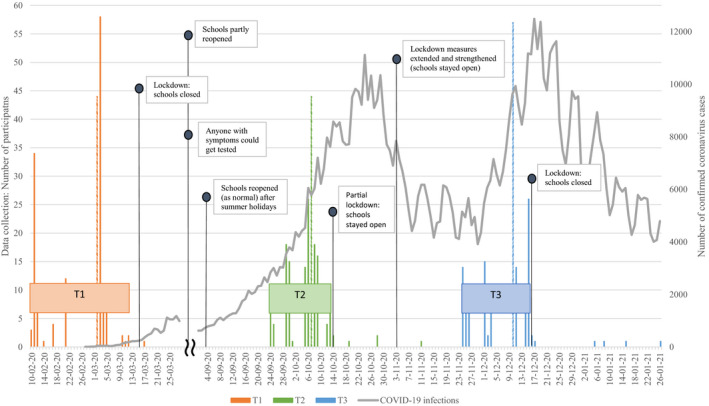
Timing of COVID‐19 pandemic in the Netherlands. *Note*. The histograms show the number of assessed participants per timepoint, for each window of data assessment (T1: orange, T2: green, and T3: blue), of which dashed lines represent the median of the data collection window. The line shows the number of confirmed coronavirus cases (i.e., infections) per day in the Netherlands (adapted from the RIVM^1^; https://www.rivm.nl/coronavirus‐covid‐19/grafieken). Please note: as from June 1, 2020, anyone with coronavirus‐symptoms could get tested (i.e., before that date corona testing capacity was restricted). Furthermore, specific government restrictions regarding school opening or closings are represented (adapted from the RIVM^1^; https://www.rivm.nl/gedragsonderzoek/tijdlijn‐maatregelen‐covid). From March 16, schools were closed. From June 2, schools partly reopened and from September, schools reopened as normal (after summer holidays). On October 14, a partial lockdown was put into effect, and from November 3, these measures were extended and strengthened (but schools stayed open). From December 16, the schools were closed again. ^1^RIVM: National Institute for Public Health and the Environment.

Pre‐COVID‐19 pandemic data were collected between February 10, 2020 and March 17, 2020, thus before the first COVID‐19 lockdown, when students were at school and unaware of the long‐lasting adversity that would come (T1). During the COVID‐19‐pandemic, data were collected at eight months after the first measurement (T2; on average after 31 weeks), when students were at school again and there were fewer restrictions. The third measurement took place two months after the second measurement (T3; on average after 9 weeks), when most students were at school and the situation deteriorated again, pending stricter restrictions. Figure 1 presents the windows of data assessment (i.e., at T1, T2, and T3), as well as the timing of COVID‐19 restrictions (e.g., opening and closing of schools) and infection rates in the Netherlands.

The sample comprised of 188 adolescents (54% female; *M*_age_ = 13.49, *SD* = .81). The participants were first‐, second‐, and third‐year students (equivalent to USA 7th, 8th, and 9th grade) in secondary schools located in one of the four largest cities in the Netherlands. The majority of the participants were in their first year of secondary school (65%) during the first measurement (T1), and in the second grade during the second (T2) and third measurement (T3). Educational level varied from (1) prevocational education level (32%), (2) prevocational or senior general education level (27%), and (3) senior general education‐preuniversity education level (41%). The second group included classes with mixed educational levels (i.e., prevocational and senior general education level). The third group included multiple educational levels, including senior education level, preuniversity education, and a combination of these two (i.e., a separate subgroup for senior general education level was not created, because this only represented 14% of the students). Further details about demographics are presented in Table 1.

**Table 1:** Demographics at Baseline (T1)

	*N*	*M* (*SD*)
Age	188	13.49 (0.81)
Number of siblings	188	1.76 (1.28)

	*N*	%
Female	102	54.3
Education level		
Prevocational education	61	32.4
Prevocational/senior general education	50	26.6
Senior general‐preuniversity education	77	41.0
School year^a^		
First year	123	65.4
Second and third year	65	34.6
Financial problems	10	5.3
Country of birth: the Netherlands	175	93.1
Ethnic identity		
Western	114	60.6
Western‐non Western	33	17.6
Non Western	41	21.8
Living situation: living with both parents	124	66.0

^a^ Equivalent to USA 7th, 8th, and 9th grade (respectively the first, second, and third year).

At T2, *N* = 10 participants were lost to follow up, and at T3, an additional *N* = 8 participants were lost to follow up (total of *N* = 18 at T3). Independent t‐tests and chi‐square tests were conducted to examine differences at T1 between participants and dropouts at T2 and T3. There were no statistically significant differences in demographics and prepandemic factors (i.e., stress, internalizing problems, coping skills, and social support) at T1 between participants and dropouts at T2 or at T3, with the exception of well‐being. Dropouts at T3 reported lower levels of well‐being at T1 than participants (*t* (185) = −2.140, *p* = .034).

Since a subset of adolescents participated in one of the skills‐training programs between T2 and T3 (51%; *N* = 96 participants were assigned to the experimental group, of which only 34 participants attended more than four sessions), we examined whether attending the skills‐training programs correlated with any of the investigated variables. Attending the skills‐training programs (i.e., number of sessions, *N* = 53 participants attended more than one session) did not correlate with demographics (i.e., gender, age, and educational level), prepandemic factors, and COVID‐19‐related concerns at T2 and T3. Hence, we did not include attending the skills‐training programs as a control variable in the analyses.

Assessments were predominantly at school, but at T1 and T3, a small number of students (T1: *N* = 1, 1%; T3: *N* = 7, 4%) were assessed online and during the lockdown (i.e., because the schools had to close suddenly). At T2, a small number of participants were assessed after the start of the partial lockdown from October 14 (*N* = 6, 3%) (See Figure 1). We performed independent t‐tests to examine whether these participants (i.e., outliers at T2 and T3) differed from the other participants in COVID‐19‐related concerns. Comparison of participants revealed no statistically significant differences, suggesting that outliers did not affect the results. Active parental and student informed consent was required for the students’ participation in the study. The study was approved by the Psychology Ethical Committee of Leiden University.

### Instruments

#### COVID‐19‐related concerns

Seven items were used to assess concerns about the COVID‐19 pandemic and the restrictions, including the possibility of getting sick, as well as concerns related to friends and school (see Table 2). *COVID‐19‐related concerns* were measured at T2 and T3 on a 5‐point scale from 1 (*never*) to 5 (*very often*). A principal component factor analysis demonstrated two distinct factors, one related to worrying about social activities (3 items; α = .77 at T2; α = .76 at T3) and the other consisting of worrying about getting sick (2 items; α = .71 at T2; α = .71 at T3). Items were averaged to create a score for the two subscales, with higher scores indicating more concerns. The two other items (worrying about school; worrying about financial problems) did not adequately fit into either factor and were analyzed as a single item. A total scale score (i.e., total COVID‐19‐related concerns) was created by averaging the seven items (7 items; α = .76 at T2; α = .78 at T3).

**Table 2:** Mean Scores for COVID‐19‐Related Concerns

	T2 (*N* = 178) *M* (*SD*)	T3 (*N* = 170) *M* (*SD*)	Paired samples t‐test (*N* = 170) *t* (*p*‐value)
How often do you worry about the following things? (1 = *never*; 5 = *very often*)			
1. That I get sick from the coronavirus.	1.95 (0.97)	2.02 (1.00)	−0.784 (.434)
2. That someone from my family or relatives get sick from the coronavirus.	2.64 (1.26)	2.62 (1.24)	0.235 (.814)
3. That I’m behind or delayed in school due to the coronavirus.	2.66 (1.29)	2.79 (1.19)	−1.112 (.268)
4. That I see my friends less because of the coronavirus.	2.64 (1.30)	2.72 (1.24)	−0.367 (.714)
5. That I can do less fun things (e.g., cinema and shopping) because of the coronavirus.	2.89 (1.34)	3.04 (1.30)	−0.988 (.325)
6. That I can no longer go to my (sports)club because of the coronavirus	2.49 (1.39)	2.42 (1.39)	0.836 (.404)
7. Financial problems for me and/or my family or relatives because of the Consequences of the coronavirus.	1.60 (1.00)	1.84 (1.11)	−2.827 (.005)
Subscale: concerns—social activities.	2.67 (1.11)	2.73 (1.07)	−0.163 (.871)
Subscale: concerns—getting sick.	2.29 (0.99)	2.32 (0.99)	−0.289 (.773)
Total scale: concerns.	2.41 (0.79)	2.49 (0.80)	−1.083 (.280)

#### Stress

The Chronic Stress Questionnaire for Children and Adolescents (CSQ‐CA) was used to measure perceived stress (de Bruin, Sieh, Zijlstra, & Meijer, 2018). Participants were asked to respond to 17 items (e.g., ‘I often get upset about things that are not important’) on a 4‐point scale ranging from 1 (*not true for me at all*) to 4 (*completely true for me*). Higher scores reflect more stress (α = .79 at T1).

#### Internalizing problems

The Dutch version of the Youth Outcome Questionnaire (Y‐OQ‐30.1) (Dunn, Burlingame, Walbridge, Smith, & Crum, 2005) was used to assess internalizing problems (i.e., anxiety and depression symptoms). A subscale was used, consisting of 6 items (e.g., ‘I am sad or unhappy’) on a 5‐point Likert scale from 0 (*never*) to 4 (*always*). Higher scores reflect more internalizing problems (α = .76 at T1).

#### Coping skills

The Dutch version of the Cognitive Emotional Regulation Questionnaire—short form (CERQ‐short) was used to measure maladaptive and adaptive coping skills (Garnefski & Kraaij, 2006). This instrument is based upon self‐report, measuring 18 items on a 5‐point Likert scale from 1 (*never*) to 5 (*always*). The maladaptive coping scale consists of the subscales self‐blame, other‐blame, rumination, and catastrophizing and comprises of 8 items (e.g., ‘I keep thinking about how terrible it is what I have experienced’). The adaptive coping consists of the subscales acceptance, refocus on planning, positive refocusing, positive reappraisal and putting into perspective, and comprises of 10 items (e.g., ‘I think I can learn something from the situation’). Higher scores reflect more maladaptive coping (α = .78 at T1) or adaptive coping (α = .79 at T1).

#### Well‐being

The Dutch version of the WHO‐Five Well‐Being Index was used to measure well‐being (WHO, 1998), consisting of 5 statements (e.g., ‘My daily life has been filled with things that interest me’). Participants were asked to think about the past two weeks and report how they felt on a 6‐point scale ranging from 0 (*at no time*) to 5 (*all of the time*). A total score was multiplied by four to create the final score, with higher scores indicating positive well‐being (α = .85 at T1).

#### Social support

The social support list—Interactions (SSL‐I) were used to measure the extent of social support adolescents received by the social interactions in their network (van Eijk, Kempen, & van Sonderen, 1994). Participants were asked to report how often a situation ever happened to them (e.g., Does it ever happen to you that people: ‘are interested in you?’ or ‘ask you for help or advice?’) on a 5‐point scale from 1 (*seldom or never*) to 4 (*very often*). A total score was created of the 12 items, with higher scores reflecting more social support (α = .92 at T1).

### Statistical analyses

Statistical analyses were conducted using SPSS version 25. To meet the first aim, descriptive analyses were performed for the COVID‐19‐related concerns, at both T2 and T3. Paired‐sample t‐tests were performed to investigate differences in COVID‐19‐related concerns between T2 and T3. In addition, gender, age (i.e., school year), and educational level (T1) differences were investigated by conducting independent t‐tests and ANOVAs. Significant ANOVAs were followed by a post hoc test. For the second aim, crude associations (correlations) between individual factors at T1 and COVID‐19‐related concerns at T2 and T3 were calculated for descriptive reasons. To examine which individual prepandemic factors predicted COVID‐19 related concerns, three‐step hierarchical regression analyses were performed for the different outcome variables, both at T2 and T3. The first step included educational level (T1) as a control variable, as educational level correlated with (subscales of) COVID‐19‐related concerns at T2. In addition, concerns at T2 were added as a covariate in the regression analyses for outcomes at T3. The second step included risk factors and consisted of stress, internalizing problems, and maladaptive coping (T1). The final third step included protective factors and consisted of adaptive coping, well‐being, and social support (T1).

## Results

### COVID‐19‐related concerns in adolescents

Table 2 presents the mean scores of COVID‐19‐related concerns reported by adolescents. The most frequently reported COVID‐19‐related concern at T2 and T3 was related to doing fewer fun things (e.g., cinema and shopping), followed by worrying about getting delayed in school, seeing friends less, and worrying that someone in the family gets sick (see Table 2). Descriptive analyses (Table 3) demonstrated that more than half of the adolescents experienced medium to high levels of COVID‐19‐related concerns about doing fewer fun things (range ‘3’ to ‘5’: 60.7%, *N* = 108 at T2 and 67.1%, *N* = 114 at T3), getting delayed in school (range ‘3’ to ‘5’: 55.6%, *N* = 99 at T2, 64.2%, *N* = 109 at T3), seeing friends less (range ‘3’ to ‘5’: 53.3%, *N* = 95 at T2 and 57.6%, *N* = 98 at T3), and someone in the family getting sick (range ‘3’ to ‘5’: 57.9%, *N* = 103 at T2 and 57.6%, *N* = 98 at T3). Concerns about financial problems were reported the least (range ‘3’ to ‘5’: 16.9%, *N* = 30 at T2 and 25.3%, *N* = 43 at T3). Differences in levels of concern from T2 to T3 were not statistically significant for any of the items and scales, except for concerns about financial problems (see Table 2).

**Table 3:** Percentages and Frequency of Responses to COVID‐19‐Related Concerns

How often do you worry about the following things?		Never % (*N*)	Seldom % (*N*)	Sometimes % (*N*)	Often % (*N*)	Very often % (*N*)
That I get sick from the coronavirus.	T2	**42.1 (75)**	27.5 (49)	24.2 (43)	5.6 (10)	0.6 (1)
	T3	**39.4 (67)**	26.5 (45)	27.6 (47)	5.3 (9)	1.2 (2)
That someone from my family or relatives get sick from the coronavirus.	T2	26.4 (47)	15.7 (28)	**33.1 (59)**	16.9 (30)	7.9 (14)
	T3	27.6 (47)	14.7 (25)	**30.0 (51)**	22.9 (39)	4.7 (8)
That I’m behind or delayed in school due to the coronavirus.	T2	26.4 (47)	18.0 (32)	**27.0 (48)**	20.2 (36)	8.4 (15)
	T3	20.0 (34)	15.9 (27)	**35.9 (61)**	21.2 (36)	7.1 (12)
That I see my friends less because of the coronavirus.	T2	**27.0 (48)**	19.7 (35)	23.0 (41)	23.0 (41)	7.3 (13)
	T3	21.8 (37)	20.6 (35)	**28.8 (49)**	21.2 (36)	7.6 (13)
That I can do less fun things (e.g., cinema, shopping) because of the coronavirus.	T2	21.9 (39)	17.4 (31)	22.5 (40)	**25.8 (46)**	12.4 (22)
	T3	17.6 (30)	15.3 (26)	26.5 (45)	**27.1 (46)**	13.5 (23)
That I can no longer go to my (sports)club because of the coronavirus	T2	**34.8 (62)**	19.7 (35)	18.0 (32)	16.9 (30)	10.7 (19)
	T2	**37.6 (64)**	17.1 (29)	21.8 (37)	12.4 (21)	11.2 (19)
Financial problems for me and/or my family or relatives because of the consequences of the coronavirus.	T2	**66.3 (118)**	16.9 (30)	9.0 (16)	6.2 (11)	1.7 (3)
	T3	**54.7 (93)**	20.0 (34)	15.9 (27)	5.9 (10)	3.5 (6)

Note

Highest percentages per item are bolded.

Tables S1–S3 present gender, age (i.e., school year), and educational level (T1) differences in COVID‐19‐related concerns (i.e., total scale and subscales). There were no statistically significant differences between males and females or age (i.e., school year) in COVID‐19‐related concerns at both T2 and T3 (see Tables S1 and S2). There was a statistically significant educational level difference in concerns about getting sick at T2, with adolescents with the highest educational level (i.e., senior general‐preuniversity education level) reporting a higher level of concerns relative to adolescents with the lowest educational level (i.e., prevocational education level). No statistically significant differences were observed at T3 (see Table S3).

### Predictors of COVID‐19‐related concerns

Correlations between the investigated variables are presented in Tables 4 and 5. Tables 6 and 7 present the hierarchical regression analyses with COVID‐19‐related concerns (i.e., total scale) and different type of concerns (i.e., social activities, getting sick, school, and financial problems) as outcome, at T2 and T3, respectively. The third step (i.e., adding protective factors) did not improve any of the models. Per outcome, the results of the final step generating a statistically significant improvement of the model are discussed.

**Table 4:** Correlations Between Individual Factors (T1) and COVID‐19‐Related Factors (T2)

	1	2	3	4	5	6	7	8	9	10	11	12	13
1. Concerns (T2)^a^	—												
2. Concerns—social activities (T2)	.847***	—											
3. Concerns—getting sick (T2)	.738***	.385***	—										
4. Concerns—school (T2)	.641***	.358***	.412***	—									
5. Concerns—financial problems (T2)	.400***	.110	.271***	.232**	—								
6. Stress (T1)	.250**	.135	.185*	.319***	.149*	—							
7. Internalizing problems (T1)	.134	−.040	.192*	.148*	.294***	.556***	—						
8. Maladaptive coping (T1)	.199**	.084	.237**	.124	.187*	.382***	.391***	—					
9. Adaptive coping (T1)	.034	.002	.110	.010	−.052	−.153*	−.247**	.319***	—				
10. Well‐being (T1)	−.131	−.013	−.142	−.164*	−.183*	−.435***	−.517***	−.139	.344***	—			
11. Social support (T1)	.008	.169*	−.119	−.103	−.148*	−.198**	−.398***	.026	.323***	.410***	—		
12. Age (T1)	.037	.014	.089	.093	−.141	.072	−.005	−.019	.159*	−.020	−.010	—	
13. Gender (T1)^b^	.043	−.001	.004	.137	.053	.107	.159*	−.006	−.106	−.128	.127	−.018	—
14. Educational level (T1)^c^	.177*	.090	.228**	.158*	.021	.046	−.130	.030	.204**	−.002	−.025	.225**	−.084

Note

***p < .001; **p < .01; *p < .05; ^a^Total scale (i.e., 7 items aggregated together); ^b^1 = male, 2 = female; ^c^1 = prevocational education level, 2 = prevocational/senior general education level, 3 = senior general‐preuniversity education level.

**Table 5:** Correlations Between Individual Factors (T1) and COVID‐19‐Related Factors (T3 and T2)

	1	2	3	4	5
1. Concerns (T3)^a^	—				
2. Concerns—social activities (T3)	.849***	—			
3. Concerns—getting sick (T3)	.740***	.382***	—		
4. Concerns—school (T3)	.703***	.446***	.482***	—	
5. Concerns—financial problems (T3)	.513***	.226**	.330***	.322***	—
6. Concerns (T2)^a^	.520***	.491***	.347***	.329***	.231**
7. Concerns—social activities (T2)	.409***	.493***	.172*	.200**	.117
8. Concerns—getting sick (T2)	.420***	.335***	.423***	.281***	.094
9. Concerns—school (T2)	.325***	.226**	.238**	.329***	.207**
10. Concerns—financial problems (T2)	.267***	.121	.209**	.171*	.442***
11. Stress (T1)	.379***	.230**	.294***	.414***	.280***
12. Internalizing problems (T1)	.187*	.049	.115	.325***	.249**
13. Maladaptive coping (T1)	.281***	.168*	.285***	.221**	.186*
14. Adaptive coping (T1)	.002	.011	.065	−.041	−.095
15. Well‐being (T1)	−.239**	−.134	−.151*	−.305***	−.222**
16. Social support (T1)	−.079	.057	−.126	−.173*	−.155*
17. Age (T1)	−.007	−.061	.070	.005	.011
18. Gender (T1)^b^	.037	.030	−.067	.131	.078
19. Educational level (T1)^c^	.094	.074	.113	.033	.025

Note

Correlations between the prepandemic factors (T1) and correlations between COVID‐19‐related concerns (T2) and prepandemic factors (T1) are already presented in Table 4 (variables 6‐19).

***p < .001; **p < .01; *p < .05; ^a^Total scale (i.e., 7 items aggregated together); ^b^1 = male, 2 = female; ^c^1 = prevocational education level, 2 = prevocational/senior general education level, 3 = senior general‐preuniversity education level.

**Table 6:** Hierarchical Regression Analyses With Types of COVID‐19‐Related Concerns as Outcome at T2 (*N *= 177)

	Variable	Concerns^a^			Social activities			Getting sick			School			Financial problems		
		Step 1	Step 2	Step 3	Step 1	Step 2	Step 3	Step 1	Step 2	Step 3	Step 1	Step 2	Step 3	Step 1	Step 2	Step 3
Educational level (T1)	β	.178*	.164*	.167*	.090	.051	.079	.228**	.245**	.220**	.158*	.138	.121	.021	.066	.062
Stress (T1)	β		.192*	.178		.199*	.176		.021	.028		.316**	.315**		−.060	−.072
Internalizing problems (T1)	β		.005	.020		−.171	−.116		.153	.136		−.008	−.030		.302**	.265*
Maladaptive coping (T1)	β		.120	.110		.071	.077		.164*	.132		.000	−.017		.092	.108
Adaptive coping (T1)	β			−.002			−.086			.103			.064			−.011
Well‐being (T1)	β			−.069			−.051			−.048			−.054			−.046
Social support (T1)	β			.091			.209*			−.062			−.039			−.026
	ΔR^2^	.032*	.070**	.007	.008	.036	.034	.052**	.076**	.010	.025*	.097***	.005	.000	.098***	.003
	*R* ^2^	.032	.102	.109	.008	.044	.078	.052	.128	.138	.025	.122	.127	.000	.099	.101
	Adjusted *R* ^2^	.026	.081	.072	.002	.022	.040	.047	.108	.102	.019	.101	.091	−.005	.078	.064

Note

β = standardized coefficient beta; ***p < .001, **p < .01; *p < .05; ^a^Total scale (i.e., 7 items aggregated together).

Step 1: educational level (covariate); Step 2: stress, internalizing problems, and maladaptive coping; level 3: adaptive coping, well‐being, and social support.

**Table 7:** Hierarchical Regression Analyses With Types of COVID‐19‐Related Concerns as Outcome at T3 (*N* = 169)

	Variable	Concerns			Social activities			Getting sick			School			Financial problems		
		Step 1	Step 2	Step 3	Step 1	Step 2	Step 3	Step 1	Step 2	Step 3	Step 1	Step 2	Step 3	Step 1	Step 2	Step 3
Concerns (T2)	β	.528***	.449***	.445***	.496***	.463***	.459***	.416***	.366***	.356***	.343***	.231**	.223**	.447***	.410***	.404***
Educational level (T1)	β	.000	−.011	−.027	.028	.010	−.002	.014	−.016	−.031	−.018	.010	−.015	.022	.016	.013
Stress (T1)	β		.250**	.229**		.159	.140		.247**	.237**		.247**	.229*		.200*	.179*
Internalizing problems (T1)	β		−.054	−.111		−.064	−.091		−.145	−.215*		.145	.107		.029	−.024
Maladaptive coping (T1)	β		.103	.115		.075	.073		.150†	.178*		.035	.019		.031	.066
Adaptive coping (T1)	β			.021			.028			.004			.077			−.041
Well‐being (T1)	β			−.111			−.104			−.042			−.142			−.059
Social support (T1)	β			−.028			.017			−.103			−.006			−.026
	ΔR^2^	.279***	.071**	.010	.250***	.028	.006	.176***	.075**	.011	.116***	.121***	.014	.201***	.052*	.006
	R^2^	.279	.350	.360	.250	.278	.284	.176	.250	.261	.116	.237	.251	.201	.253	.259
	Adjusted R^2^	.270	.330	.328	.241	.256	.249	.166	.227	.224	.105	.213	.213	.191	.230	.222

Note

β = standardized coefficient beta; ***p < .001, **p < .01; *p < .05; †*p* = .052; ^a^Total scale (i.e., 7 items aggregated together).

Step 1: Concerns at T2 and educational level (covariates); Step 2: stress, internalizing problems, and maladaptive coping; level 3: adaptive coping, well‐being, and social support.

At T2, for COVID‐19‐related concerns (i.e., total scale), the second step improved the model (explaining an additional 7.0% of the variance), which accounted for a total of 10.2% of variance (*F* = 4.879, *p* < .01). Prepandemic stress was a statistically significant predictor of total COVID‐19‐related concerns. For COVID‐19‐related concerns about social activities, none of the steps were statistically significant, indicating that none of the prepandemic factors were associated with concerns related to social activities. For COVID‐19‐related concerns about getting sick, the second step improved the model (explaining an additional 7.6% of the variance), which accounted for a total of 12.8% of the variance (*F* = 6.325, *p* < .001). Prepandemic maladaptive coping was a statistically significant predictor of concerns related to getting sick. For COVID‐19‐related concerns about school, the second step improved the model (explaining an additional 9.7% of the variance), which accounted for a total of 12.2% of the variance (*F* = 5.953, *p* < .001). Prepandemic stress was a statistically significant predictor of concerns related to school. For COVID‐19‐related concerns about financial problems, the second step improved the model (explaining an additional 9.8% of variance), which accounted for a total of 9.9% of the total variance (*F* = 4.698, *p* < .01). Levels of prepandemic internalizing problems were a statistically significant predictor of concerns related to financial problems (See Table 6).

At T3, for COVID‐19‐related concerns (i.e., total scale), the second step improved the model (explaining an additional 7.1% of the variance), which accounted for a total of 35.0% of variance (*F* = 17.540, *p* < .001). Prepandemic stress was a statistically significant predictor of total concerns. For COVID‐19‐related concerns about social activities, adding the prepandemic risk factors did not improve the model, indicating that none of the factors predicted concerns related to social activities. For COVID‐19‐related concerns about getting sick, the second step improved the model (explaining an additional 7.5% of the variance), which accounted for a total of 25.0% of variance (*F* = 10.888, *p* < .001). Prepandemic stress was a statistically significant predictor of concerns related to getting sick, as was maladaptive coping at *p* = .052. For COVID‐19‐related concerns about school, the second step improved the model (explaining an additional 12.1% of the variance), which accounted for a total of 23.7% of the variance (*F* = 10.111, *p* < .001). Prepandemic stress was a statistically significant predictor of concerns related to school. For COVID‐19‐related concerns about financial problems, the second step improved the model (explaining an additional 5.2% of variance), which accounted for a total of 25.3% of the variance (*F* = 11.036, *p* < .001). Again, prepandemic stress was a statistically significant predictor of concerns related to financial problems (see Table 7).

In sum, prepandemic stress predicted total COVID‐19‐related concerns and concerns about school at both T2 and T3, and predicted concerns about getting sick and financial problems at T3. Prepandemic maladaptive coping predicted concerns about getting sick at both T2 and T3 and prepandemic internalizing problems predicted concerns about financial problems at T2. None of the prepandemic factors were associated with concerns about social activities (at both T2 and T3). Protective factors did not predict total COVID‐19‐related concerns nor specific concerns (i.e., social activities, getting sick, school, and financial problems) at T2 and T3.

## Discussion

The present study aimed to describe COVID‐19‐related concerns in adolescents, and gender, age, and educational level differences in reported concerns. Furthermore, we aimed to investigate which individual prepandemic risk and protective factors predicted COVID‐19‐related concerns at eight and ten months into the pandemic. Results demonstrated that more than half of the adolescents experienced medium to high levels of COVID‐19‐related concerns at both stages during the pandemic. Adolescents most frequently expressed concerns about restricted possibilities to do fun things, but also reported concerns about getting delayed in school, seeing friends less, and someone in the family getting sick. Adolescents were least concerned about financial problems, followed by getting sick themselves. The level of COVID‐19‐related concerns remained stable from eight to ten months into the pandemic, with the exception of concerns about financial problems, which increased over time. Eight months into the pandemic, adolescents with the highest educational level reported more concerns about getting sick (i.e., self or family) than adolescents with the lowest educational level. There were no other educational level differences nor any gender or age differences in expressed concerns. Prepandemic predictors of COVID‐19‐related concerns differed somewhat depending on type of concern. Eight months into the pandemic, total COVID‐19‐related concerns and concerns about school were predicted by prepandemic stress, while concerns about getting sick were predicted by prepandemic maladaptive coping and concerns about financial problems were predicted by prepandemic internalizing problems. Ten months into the pandemic, total COVID‐19‐related concerns, as well as concerns about getting sick, school, and financial problems were predicted by prepandemic stress. In addition, concerns about getting sick were predicted (at *p* = .052) by prepandemic maladaptive coping.

More than half of the adolescents reported medium to high levels of COVID‐19‐related concerns at both stages during the pandemic, with an overall (nonsignificant statistical) trend for higher levels of concern over the course of the pandemic. The results of the present study suggest that adolescents may experience continued and possibly increased COVID‐19‐related concerns during the course of the pandemic. It is plausible that this is related to the long‐lasting and persistent government measures and restrictions, the unpredictability of the (course of the) pandemic, and the increased uncertainty about the future. Further research at multiple timepoints is necessary to gain more knowledge about the changes over the course of the pandemic. Nevertheless, seasonal effects should be taken into account, as recent research demonstrated that youth reported more depressive symptoms during the winter months (Lukmanji, Williams, Bulloch, & Patten, 2020), which can distort results.

Overall, adolescents most frequently expressed concerns about social activities and getting delayed in school at both stages during the COVID‐19 pandemic. In contrast, adolescents expressed the least concerns about financial problems and getting sick themselves. These findings are in line with previous research in adolescents at the beginning of the COVID‐19 pandemic (Ellis et al., 2020; Magson et al., 2020; Muñoz‐Fernández & Rodríguez‐Meirinhos, 2021; Ravens‐Sieberer et al., 2021). This combination of findings suggests that concerns about social activities and school persist throughout the pandemic, possibly due to the long‐lasting and persistent government measures and restrictions. Governments should be aware of the negative consequences of the restrictions (e.g., social distancing), and may want to stimulate alternative online social activities for this age group, as digital connectedness might attenuate the negative effects of social deprivation in adolescents (Orben, Tomova, & Blakemore, 2020). Furthermore, concerns about financial problems, the least reported concern in the current study, increased during the COVID‐19 pandemic. It is plausible that some adolescents and their families experience more negative financial consequences as the pandemic continues, because of the economic consequences of the long‐lasting, strict, and persistent government measures (e.g., closing of restaurants and non‐essential shops). Families with financial struggles should receive attention and support, as the negative economic consequences will presumably deteriorate throughout the pandemic, and may persist in the long term. Remarkably, adolescents reported that they were not very concerned about getting sick themselves. This may be explained by the lower level of risk appraisal that characterizes adolescence. For instance, as levels of risk‐taking behavior, impulsivity, seeking out novel experiences, and sensation seeking are highest during adolescence (i.e., around 14–16 years; Collado, Felton, MacPherson, & Lejuez, 2014; Harden & Tucker‐Drob, 2011), it could be that adolescents feel less susceptible to infection by COVID‐19 and thus reported few concerns about getting sick. Moreover, it is possible that as adolescents generally have mild symptoms after COVID‐19 infection and generally have a good prognosis (Mantovani et al., 2021), they worry less about getting sick themselves.

The results demonstrated that eight months into the pandemic, adolescents with a higher educational level experienced more COVID‐19‐related concerns related to getting sick than adolescents with a lower educational level, whereas no educational differences were found ten months into the pandemic. Given their initial lower level of concern about getting sick, adolescents with lower educational levels may have needed more time to grasp the impact of the pandemic on their own and families’ health, and therefore reported fewer concerns about getting sick at the first measurement during the pandemic. Alternatively, as T2 was in a period of relatively few government restrictions (i.e., restrictions were somewhat lifted, see Figure 1), adolescents with higher educational levels might have anticipated the upcoming lockdown and accompanying stricter restrictions, reflecting more concerns at the first measurement, while adolescents with lower educational levels did not.

The results demonstrate that adolescents with specific vulnerabilities (i.e., higher stress, maladaptive coping, or internalizing problems) before the pandemic experience higher levels of concerns during the COVID‐19 pandemic. The finding that prepandemic stress is an important and persistent predictor of total and specific COVID‐19‐related concerns (i.e., getting sick, school, and financial problems) suggests that stress is an important general and broad risk factor for concerns during the COVID‐19 pandemic. This is in line with a previous study in adults which observed that prepandemic stress predicted higher levels of COVID‐19‐related burden at the beginning of the pandemic (Brailovskaia & Margraf, 2020). As stress about school is one of the most reported types of stress among adolescents, including stress related to tests, grades, homework, and the future (de Anda et al., 2000), it is likely that adolescents with higher prepandemic stress are more concerned about getting delayed in school during the COVID‐19 pandemic (at both assessment points). Moreover, as the lives of adolescents changed drastically during the COVID‐19 pandemic, particularly adolescents’ school life, it is plausible that adolescents who are already stressed before the pandemic, also have more COVID‐19‐related concerns about school. Interestingly, prepandemic stress also predicted higher concerns related to getting sick and financial problems, but only at ten months into the pandemic. Possibly, the negative effects of prepandemic stress on these concerns became apparent only later in the pandemic because of the continued restrictions and uncertainty about the future, the unpredictability, the possible increased feelings of hopelessness, and the prospect of another (stricter) lockdown. Overall, adolescents with higher levels of prepandemic stress appear a vulnerable group in terms of COVID‐19‐related concerns, placing them at risk for adverse developmental outcomes at a later stage, such as school and mental health problems (Wade et al., 2020). This specific group of adolescents requires extra attention and support throughout and after the pandemic, to help them cope with the changed (social) situation.

Remarkably, maladaptive coping before the pandemic predicted higher levels of COVID‐19‐related concerns about getting sick (i.e., getting sick themselves or a relative getting sick), not about other types of concerns. Possibly, adolescents who use more negative ways to cope with stressful situations may view the risk of themselves or a relative becoming seriously ill as the most unpredictable and uncontrollable, as it appears random who gets infected and sick or not. Moreover, this type of concern might be seen by some, potentially including adolescents with maladaptive coping, as most serious, as many people became ill or died from the virus.

Prepandemic internalizing problems (i.e., anxiety and depression symptoms) were predictive of COVID‐19‐related concerns about financial problems, but only at eight months into the pandemic. It could be that adolescents with high levels of internalizing problems are especially prone to concerns about financial problems, as these concerns are specific, forceful, and family oriented, possibly reflecting more problems than only financial ones (e.g., parental conflicts or single‐parent families). Previous research demonstrated that high perceived familial financial stress was related to internalizing problems in young adolescents (Liu & Merritt, 2018). Therefore, it could also be that because of existing family financial problems, adolescents develop internalizing problems, which aggravated during the COVID‐19 pandemic due to the restrictions and accompanied economic consequences. Hence, adolescents with internalizing problems reported more concerns about financial problems. These findings indicate that adolescents and families that experience high financial stress need attention and support, as internalizing problems in adolescents and financial problems are related, which may become more pressing as the pandemic continues (e.g., because of prolonged closing of restaurants, shops).

Interestingly, all prepandemic predictors of COVID‐19‐related concerns were risk factors, including stress, maladaptive coping, and internalizing problems. Protective factors before the pandemic did not seem to affect the extent of COVID‐19‐related concerns. This contrasts previous research, which demonstrated that positive mental health predicted lower levels of COVID‐19‐related burden in adults (Brailovskaia & Margraf, 2020). However, this is in line with the notion that bad is stronger than good, suggesting that it is evolutionary adaptive to respond more strongly to bad events than good ones, which makes it more likely to survive threats (Baumeister, Bratslavsky, Finkenauer, & Vohs, 2001). In addition, as the COVID‐19 pandemic is a unique situation, linked to negative associations such as hopelessness, uncertainty, unpredictability, and mental health problems, it is plausible that only risk factors significantly predicted COVID‐19‐related concerns. Nevertheless, some adolescents might benefit from the societal changes caused by the lockdown and government restrictions (Dvorsky et al., 2020). Indeed, previous research showed that young adolescents reported slightly decreased problem behavior and in general low stress at the beginning of the COVID‐19 pandemic, as well as more free time and time with their family, probably serving as protective factors (Achterberg et al., 2021). This indicates that not all adolescents experience negative effects of the pandemic. Future research should therefore not only focus on the negative effects of the pandemic, but also on potential positive or adaptive effects.

### Limitations

The first limitation of the current study is that only seven items regarding COVID‐19‐related concerns were used, reflecting some but not all important areas of possible concerns. Even though the two subscales we used had adequate internal consistency, they consisted of only a few items. Furthermore, two additional items were analyzed separately. In addition, although the items to assess COVID‐19‐related concerns can be distinguished from items assessing the prepandemic factors by their explicit link with the coronavirus (see Table 3), we cannot rule out the possibility that part of the variance is attributable to overlap between measurement methods (i.e., common method variance). The findings of this study should, therefore, be interpreted with caution. However, we provided a first insight in the extent of COVID‐19‐related concerns adolescents experienced at eight and ten months into the COVID‐19 pandemic.

Second, the adolescents in this study were enrolled in an evaluation study investigating the effectiveness of two school‐based skills‐training programs (i.e., short prevention programs offered in schools to adolescents who expressed interest in participating in the program). Although this selection may have affected the representativeness of the sample to the (Dutch) adolescent population, our sample was comparable to the Dutch population of 10 to 15 year olds in terms of minority background (CBS, 2021a) and educational level (CBS, 2021b). In addition, prepandemic scores on stress and well‐being were generally comparable to levels of stress and well‐being in community samples of adolescents (de Bruin et al., 2018; McMahon et al., 2017). Furthermore, as only few adolescents attended one or more program sessions and attending sessions did not correlate with COVID‐19‐related concerns, we believe our findings are generalizable to a broad group of (Dutch) adolescents.

Finally, interpreting the findings in relation to government restrictions is complicated, as the measurements were not assessed prior to or after specific government measures, and restrictions were subject to frequent changes. For instance, the first measurement during the pandemic (i.e., T2) was after the summer holidays, not immediately after the first lockdown. In addition, since there was limited time between the assessments during the COVID‐19 pandemic (i.e., 9 weeks), it is possible that the assessments do not represent distinct COVID‐19 phases. However, as shown in Figure 1, the situation in the Netherlands was worse in December than in October in terms of restrictions (i.e., more and stricter restrictions) and infection rates (i.e., more infections), indicating that the two data assessments can be seen as distinct COVID‐19 phases.

### Implications

The current study showed that adolescents reported most concerns about social activities and school during the COVID‐19 pandemic, with an overall trend for higher levels of concerns over the course of the pandemic. Following this trend, it is plausible that in the postpandemic situation, adolescents are still concerned about their social network and academic delay. In order to help adolescents catch up, it is important to organize social activities such as school outings and gatherings. Moreover, adolescents should be encouraged to ask for guidance and support from their social network (i.e., peers, teachers, and parents). Further, to prevent school delays and adverse (school) outcomes, governments should fund schools to detect at‐risk adolescents and develop a personalized support plan. It is crucial that adolescents feel supported and know where to go for help and guidance.

Additionally, since specific vulnerabilities before the pandemic resulted in more concerns during the COVID‐19 pandemic, it is important to screen and monitor adolescents with heightened levels of distress (i.e., stress, maladaptive coping, and internalizing problems), as these adolescents may be at risk for later adverse developmental outcomes. Vulnerable adolescents may benefit from (early) intervention programs to alleviate stress or internalizing symptoms. Therefore, access to mental health services should be facilitated. For instance, schools might be particularly suitable for intervention programs, as previous research demonstrated that school‐based intervention programs have the potential to reduce psychosocial stress, anxiety, and depression, in particular for at‐risk adolescents (Feiss et al., 2019; van Loon et al., 2020). Furthermore, online mental health interventions also show potential in promoting adolescents’ mental health (Clarke et al., 2015), which is promising considering the possible long‐term changes in adolescents’ social lives as a consequence of the COVID‐19 pandemic.

## Conclusion

Adolescents with high levels of concern during the COVID‐19 pandemic may be at risk for later adverse developmental outcomes, such as school and mental health problems. In order to identify and help these adolescents, the current study aimed to investigate the extent of COVID‐19‐related concerns in adolescents at eight and ten months into the COVID‐19 pandemic. Moreover, we examined its relation with prepandemic risk and protective factors. Adolescents most frequently reported concerns about social activities and getting delayed in school, at two stages during the pandemic. An important finding was that adolescents who have specific vulnerabilities before the pandemic (i.e., higher stress, maladaptive coping, or internalizing problems), experience more concerns during the pandemic, placing them at risk for potential long‐term negative effects of the pandemic. These vulnerable adolescents need guidance and support as soon as possible, in order to prevent later adverse developmental outcomes.

## Supporting information

**Table S1**. Gender (T1) Differences in COVID‐19‐Related Factors at T2 and T3
**Table S2**. Age (i.e., School Year; T1) Differences in COVID‐19‐Related Factors at T2 and T3
**Table S3**. Educational Level (T1) Differences in COVID‐19‐Related Factors at T2 and T3
