# Supplementary material for: Prepandemic Risk Factors of COVID‐19‐Related Concerns in Adolescents During the COVID‐19 Pandemic
Source: J Res Adolesc. 2021 Aug 26;31(3):531–45. doi: 10.1111/jora.12651 (PMC8456903; doi:10.1111/jora.12651)
Supplement: Supplementary file 1 — Table S1. Gender (T1) Differences in COVID‐19‐Related Factors at T2 and T3 Table S2. Age (i.e., School Year; T1) Differences in COVID‐19‐Related Factors at T2 and T3 Table S3. Educational Level (T1) Differences in COVID‐19‐Related Factors at T2 and T3 [file JORA-31--s001.docx]

Supplementary Table 1.

*Gender (T1) Differences in COVID-19-Related Factors at T2 and T3*

|  | T2 |  |  | T3 |  |  |
| --- | --- | --- | --- | --- | --- | --- |
|  | Males (*N* = 81)  *M* (*SD*) | Females (*N* = 97)  *M* (*SD*) | *t-*value (*p*) | Males (*N* = 77)  *M* (*SD*) | Females (*N* = 93)  *M* (*SD*) | *t*-value (*p*) |
| Concerns | 2.37 (0.78) | 2.44 (0.80) | -0.573 (.568) | 2.46 (0.82) | 2.52 (0.79) | -0.475 (.636) |
| Concerns – social activities | 2.67 (1.15) | 2.67 (1.08) | 0.008 (.994) | 2.69 (1.09) | 2.76 (1.07) | -0.384 (.702) |
| Concerns – getting sick | 2.29 (1.00) | 2.30 (0.99) | -0.059 (.953) | 2.40 (1.04) | 2.26 (0.95) | 0.868 (.387) |
| Concerns – school | 2.47 (1.30) | 2.82 (1.27) | -1.840 (.067) | 2.62 (1.19) | 2.94 (1.18) | -1.710 (.089) |
| Concerns – financial problems | 1.54 (0.94) | 1.65 (1.05) | -0.706 (.481) | 1.74 (1.06) | 1.91 (1.16) | -1.013 (.313) |

Supplementary Table 2.

*Age (i.e., School Year; T1) Differences in COVID-19-Related Factors at T2 and T3*

|  | T2 |  |  | T3 |  |  |
| --- | --- | --- | --- | --- | --- | --- |
|  | Year 1 (*N* = 117)  *M* (*SD*) | Year 2-3 (*N* = 61)  *M* (*SD*) | *t*-value (*p*) | Year 1 (*N* = 112)  *M* (*SD*) | Year 2-3 (*N* = 58)  *M* (*SD*) | *t-*value (*p*) |
| Concerns | 2.39 (0.82) | 2.46 (0.72) | -0.559 (.577) | 2.42 (0.80) | 2.63 (0.80) | -1.604 (.111) |
| Concerns – social activities | 2.66 (1.09) | 2.70 (1.15) | -0.219 (.827) | 2.68 (1.08) | 2.82 (1.06) | -0.774 (.440) |
| Concerns – getting sick | 2.22 (1.05) | 2.43 (0.84) | -1.459 (.147) | 2.23 (0.98) | 2.50 (1.00) | -1.679 (.095) |
| Concerns – school | 2.61 (1.36) | 2.77 (1.15) | -0.846 (.399) | 2.72 (1.22) | 2.93 (1.12) | -1.079 (.282) |
| Concerns – financial problems | 1.68 (1.07) | 1.46 (0.83) | 1.489 (.139) | 1.73 (1.11) | 2.03 (1.11) | -1.688 (.093) |

Supplementary Table 3.

*Educational Level (T1) Differences in COVID-19-Related Factors at T2 and T3*

|  | T2 | | | | T3 | | | |
| --- | --- | --- | --- | --- | --- | --- | --- | --- |
|  | Prevocational (*N* = 57) | Prevocational / senior general (*N* = 46) | Senior general-preuniversity (*N* = 75) |  | Prevocational (*N* = 54) | Prevocational / senior general (*N* = 44) | Senior general-preuniversity (*N* = 72) |  |
|  | *M* (*SD*) | *M* (*SD*) | *M* (*SD*) | *F*-value (*p*) | *M* (*SD*) | *M* (*SD*) | *M* (*SD*) | *F*-value (*p*) |
| Concerns | 2.24 (0.85) | 2.39 (0.81) | 2.56 (0.70) | 2.848 (.061) | 2.39 (0.90) | 2.49 (0.77) | 2.57 (0.74) | 0.753 (.473) |
| Concerns – social activities | 2.57 (1.07) | 2.61 (1.14) | 2.80 (1.12) | 0.793 (.454) | 2.60 (1.15) | 2.78 (1.10) | 2.79 (1.00) | 0.567 (.568) |
| Concerns – getting sick | 1.96 (0.99) | 2.39 (1.10) | 2.49 (0.85) | 5.443 (.006)^a^ | 2.18 (0.99) | 2.32 (1.08) | 2.44 (0.93) | 1.075 (.344) |
| Concerns – school | 2.42 (1.38) | 2.59 (1.24) | 2.89 (1.24) | 2.302 (.103) | 2.78 (1.28) | 2.70 (1.21) | 2.86 (1.12) | 0.241 (.786) |
| Concerns – financial problems | 1.61 (1.08) | 1.50 (0.98) | 1.65 (0.95) | 0.340 (.712) | 1.83 (1.27) | 1.75 (1.08) | 1.89 (1.01) | 0.211 (.810) |

^a^Welch’s *F* and significant post-hoc test between prevocational education level and senior general education-preuniversity education level.
